# Clinical Diagnosis of Cytomegalovirus Colitis Enhanced With Multiplex Polymerase Chain Reaction Panel Using Stool Sample: A Case Report

**DOI:** 10.7759/cureus.64986

**Published:** 2024-07-20

**Authors:** Shinobu Hosokawa, Akihiro Bessho, Mai Kagawa, Masahiro Oda, Makoto Sakugawa

**Affiliations:** 1 Department of Respiratory Medicine, Japanese Red Cross Okayama Hospital, Okayama, JPN; 2 Department of Clinical Laboratory, Japanese Red Cross Okayama Hospital, Okayama, JPN

**Keywords:** cmv pcr, stool, multiplex pcr, immunosuppression, cytomegalovirus colitis

## Abstract

A 71-year-old man was admitted because of acute exacerbation of interstitial pneumonia following a right upper lobectomy for lung cancer. His respiratory failure worsened after admission, and he required mechanical ventilation. He was undergoing intensive immunosuppressive treatment, including high-dose corticosteroids and cyclosporine, and had watery diarrhea six times a day. White blood cells were found in the stool, and an intestinal infection was suspected. Fecal cultures showed no pathogenic bacteria. Multiplex polymerase chain reaction for gastrointestinal infection yielded negative results. Based on the increasing number of cytomegalovirus (CMV) antigen-positive cells in the CMV antigenemia assay, we suspected CMV colitis. However, the patient was still undergoing mechanical ventilation, and colonoscopy was difficult to perform. After explaining the procedure to the patient and obtaining his consent, the BioFire® FilmArray® Meningitis/Encephalitis (ME) Panel was performed using a fecal specimen. CMV was detected. Intravenous infusion of ganciclovir at 5 mg/kg was immediately commenced and administered every 12 hours for three weeks. Intravenous infusion at 5 mg/kg was continued every 24 hours thereafter for a further three weeks. When CMV colitis is suspected but the patient’s condition prevents tissue collection through colonoscopy and standard diagnosis by histopathology, the addition of CMV PCR using a stool sample may assist in the clinical diagnosis of CMV colitis. The use of multiplex polymerase chain reaction is expected to contribute to prompt and appropriate treatment.

## Introduction

Human cytomegalovirus (CMV) is a ubiquitous species-specific herpes virus. CMV infection is very common and is mostly subclinical, remaining latent in myelomonocytic cells throughout life. The latent virus is reactivated when the host becomes immunosuppressed or immunocompromised, resulting in the development of CMV disease [1]. Inflammatory bowel disease (IBD) is also associated with an increased risk of developing CMV colitis. The large intestine is one of the end organs affected by CMV reactivation. The diagnosis of CMV colitis classically necessitates the presence of clinical symptoms, endoscopic mucosal findings, and pathological evidence of CMV, including hematoxylin and eosin (HE) staining and immunohistochemical (IHC) staining [2]. Although histopathologic diagnosis may be the most specific approach, endoscopic examination is an invasive procedure. For example, patients who develop CMV colitis are often in poor general condition, which makes the performance of endoscopic examination challenging. In addition, in patients with weakened colon walls due to IBD, complications, such as bleeding or intestinal perforation, during colonoscopy are a concern. Furthermore, obtaining the result of histological examination takes time. The real-time polymerase chain reaction (PCR) assay has been introduced as a rapid test for the detection of pathogens. The multiplex PCR system has become increasingly prevalent in recent years, enabling simultaneous testing for multiple pathogens, including bacteria, viruses, and yeast, as well as antimicrobial resistance genes in some cases. We herein report our experience in which a multiplex PCR assay of a stool sample was useful for the diagnosis of CMV colitis in a patient for whom endoscopic diagnosis was difficult.

## Case presentation

**Figure 1:**
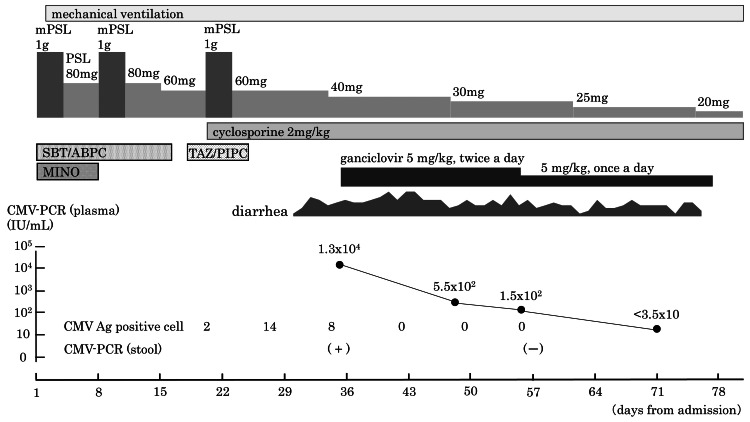
Clinical course CMV Ag, cytomegalovirus antigen; CMV-PCR, cytomegalovirus-polymerase chain reaction; MINO, minocycline hydrochloride; mPSL, methylprednisolone; PSL, prednisolone; SBT/ABPC, sulbactam sodium/ampicillin sodium; TAZ/PIPC, tazobactam/piperacillin hydrate

A 71-year-old man was admitted to our hospital because of acute exacerbation of interstitial pneumonia 10 days after undergoing a right upper lobectomy for lung cancer. He had comorbid conditions, including diabetes, hypertension, and hyperlipidemia, which had been well controlled with medication. The day after hospitalization, the patient’s respiratory failure worsened, necessitating the initiation of mechanical ventilation. Despite the administration of intravenous methylprednisolone pulse therapy (1 g/day × 3 days) twice and high-dose prednisolone (1 mg/kg/day), the interstitial pneumonia did not improve. Three weeks after admission, the patient commenced treatment with the immunosuppressive drug cyclosporine in conjunction with a third round of methylprednisolone pulse therapy. Given that the patient had been undergoing intensive immunosuppressive treatment, screening for CMV antigen was initiated concurrently. A CMV antigenemia assay using the monoclonal antibody C7-HRP demonstrated the presence of two positive cells per 50,000 cells. The result of the CMV IgG test was positive, while that of the CMV IgM test was negative. One week later, the CMV antigenemia assay demonstrated 14 positive cells. The patient subsequently developed watery diarrhea six times a day. His white blood cell count was 12,250/μL, and his C-reactive protein concentration was 3.49 mg/dL, both of which were slightly elevated. The presence of white blood cells in the stool indicated the possibility of an intestinal infection. Fecal cultures yielded no pathogenic bacteria. The BioFire® FilmArray® Gastrointestinal Panel was performed using a stool sample, but no pathogens were detected. A diagnosis of CMV colitis was suspected based on the increasing number of CMV antigen-positive cells in the antigenemia assay. However, the patient was still undergoing mechanical ventilation, and a colonoscopy for diagnosis of CMV colitis by histology would have been difficult. Once the patient had been informed that the test would be performed using a non-designated specimen and he had consented to the procedure, the BioFire® FilmArray® Meningitis/Encephalitis (ME) Panel was performed using a stool sample. This revealed the presence of CMV. *Escherichia coli* was also detected in the patient’s stool sample. A 5-mg/kg intravenous infusion of ganciclovir was initiated every 12 hours and continued for three weeks. A 5-mg/kg intravenous infusion was continued every 24 hours thereafter for a further three weeks. The patient’s diarrhea gradually improved. Regarding changes in CMV testing results during the course of treatment, immediately prior to the commencement of ganciclovir treatment, the CMV antigenemia assay demonstrated the presence of eight positive cells, while the quantitative value of CMV nucleic acid in plasma was 1.3 × 10^4^ IU/mL. One week after commencing ganciclovir treatment, the CMV antigenemia assay (C7-HRP) demonstrated a negative result. The quantitative value of CMV nucleic acid in plasma decreased to 5.5 × 10^2^ IU/mL two weeks after the commencement of treatment and 1.5 × 102 IU/mL three weeks after the commencement of treatment. Three weeks after commencing treatment, the BioFire® FilmArray® ME Panel was repeated using feces, and no evidence of CMV was detected (Figure 1).

## Discussion

In this case, an immunosuppressed patient presented with frequent diarrhea. The number of CMV antigen-positive cells was high, leading to suspicion of CMV colitis. However, obtaining a pathological diagnosis using tissue specimens collected via colonoscopy, which is the conventional standard diagnostic method, would have been difficult for this patient. To enhance the diagnostic specificity, a CMV PCR test was conducted using a stool sample.

In the diagnosis of end-organ CMV disease, such as CMV colitis, pathological evidence of CMV (including HE and IHC staining) has been the diagnostic standard. Although pathological examination has high specificity (92%-100%), it has low sensitivity (10%-87%) [3-5]. In recent years, the detection and quantification of CMV have become possible through the use of PCR. CMV reactivation in immunosuppressed patients is monitored by quantitative PCR using blood samples in a manner analogous to antigenemia [6,7]. Moreover, the European Crohn’s and Colitis Organisation recommends that obtaining colonic tissue for IHC, PCR, or both is essential for confirming active CMV colitis in patients with IBD and should be the standard technique [8]. However, a colonoscopy is required to collect tissue samples for histological examination, and this can be extremely invasive for severely ill patients. Consequently, the focus shifted to CMV PCR using feces. The methodology and outcomes of CMV PCR using stool samples from immunocompromised patients were first reported in 1995 [9]. Several studies utilizing stool CMV PCR as a diagnostic method for CMV colitis were subsequently conducted, with sensitivity ranging from 16.7% to 84.7% and specificity ranging from 71.4% to 96.0% [10]. Specimens from 117 patients in whom CMV enteritis was clinically suspected, and tissues were collected using colonoscopy were subjected to histological examination (HE and IHC staining), CMV PCR of colonic mucosal tissues, CMV PCR of stool samples, and CMV PCR of plasma [10]. When cytomegalic cells were detected by HE or IHC staining as the reference standard for the diagnosis of CMV colitis, the sensitivity and specificity of stool CMV PCR were found to be 70.4% and 91.6%, respectively. The combination of positive stool CMV PCR and positive plasma CMV PCR was found to increase the specificity to 96% with a 79% positive predictive value. Consequently, it was recommended that in the absence of a colonoscopy, the commencement of treatment for CMV colitis should be considered in the event of a positive result from both tests. In our case, the plasma quantitative CMV PCR was performed immediately before the start of treatment, and the result was positive. However, the plasma CMV PCR result was obtained after the start of treatment because the treatment had been already started based on the results of other tests.

The PCR method subsequently became a more familiar test, and multiplex PCR came to be widely applied for infectious diseases. The use of multiplex PCR systems for the detection of pathogens in infectious diseases has expanded beyond research institutions to encompass general hospitals, where these systems facilitate rapid diagnosis and treatment in clinical settings. Several multiplex PCR kits are available for the detection of pathogens associated with gastrointestinal infections. However, none of these kits include CMV PCR. Multiplex PCR kits for pathogens associated with meningitis and encephalitis include CMV PCR. One study evaluated a multiplex PCR assay for the detection of CMV in stool samples from patients with ulcerative colitis [11]. The Seeplex Meningitis V1 ACE detection kits (Seegene, Seoul, South Korea) were employed to detect CMV from stool samples, and CMV was identified in 37 of 300 patients (12.3%).

We have adopted the BioFire® FilmArray® Torch system at our institution, and we perform various BioFire® FilmArray® panel tests in our daily clinical practice. Once the basic tests had been completed, a multiplex PCR test was performed on non-designated specimens to exceed the current diagnostic limits. Using stool samples for CMV PCR for the detection of CMV colitis is underreported. One limitation is that CMV PCR using stool samples is not sensitive enough. The other limitation is that multiplex PCR using stool samples has not been verified and validated in the ME Panel. Although CMV is less common than the causative pathogens of most gastrointestinal infections, it is expected to be included in the gastrointestinal panel, given the severity of CMV disease and the specificity of its treatment.

## Conclusions

In conclusion, if a diagnosis of CMV colitis is suspected but colonoscopy is not possible and a standard diagnosis cannot be made, the addition of CMV PCR using a stool sample may assist in the clinical diagnosis of CMV colitis. The use of multiplex PCR panel testing is expected to facilitate prompt and appropriate treatment. In the present case, CMV was not included in the target pathogens of the multiplex PCR panel for gastrointestinal infections. Therefore, we devised a method in which we used non-designated fecal specimens for a meningitis/encephalitis panel containing CMV. The limitations are that CMV PCR using stool samples is not sensitive enough and that multiplex PCR on non-designed samples is not verified and validated.
